# Risk Factors for Barotrauma with Extra-Alveolar Air in a Selected COVID-19 Patient Population: Experience from a Tertiary University Hospital

**DOI:** 10.3390/jcm15093422

**Published:** 2026-04-30

**Authors:** Jian Hai Chai, Azlina Masdar, Aliza Mohamad Yusof, Nadia Md Nor, Rufinah Teo, Iskandar Khalid, Wan Rahiza Wan Mat

**Affiliations:** 1 Department of Anaesthesiology and Intensive Care, Faculty of Medicine, Universiti Kebangsaan Malaysia, Cheras, Kuala Lumpur 56000, Malaysia; jianhai724@hctm.ukm.edu.my (J.H.C.); alizamyusof@gmail.com (A.M.Y.); nadiamn@hctm.ukm.edu.my (N.M.N.); rufinah@hctm.ukm.edu.my (R.T.); docaweng@yahoo.com (W.R.W.M.); 2 Department of Anaesthesiology and Intensive Care, Hospital Canselor Tuanku Muhriz, Jalan Yaacob Latif, Bandar Tun Razak, Cheras, Kuala Lumpur 56000, Malaysia; iskandarkhalid@gmail.com

**Keywords:** ARDS, vaccine, mechanical ventilation, organizing pneumonia, pulmonary, barotrauma

## Abstract

**Background/Objectives**: Mechanical ventilation (MV) is a crucial intervention in managing severe respiratory failure due to COVID-19. However, its use may be complicated by pulmonary barotrauma, a serious event associated with increased morbidity and mortality. Understanding its incidence and associated risk factors is essential for optimising ventilatory strategies and improving patient outcomes. The aim of this study was to determine the incidence and risk factors associated with the development of pulmonary barotrauma in mechanically ventilated patients with COVID-19. **Methods**: All mechanically ventilated patients aged 18 years and above who were admitted to the COVID-19 Intensive Care Unit (ICU) from January 2021 to June 2022 were included. Patients who developed pulmonary barotrauma prior to or within 24 h of ICU admission, had iatrogenic pneumothorax, were readmitted to the ICU, or were ventilated for causes other than COVID-19-related respiratory failure were excluded. Data on patient demographics, vaccination status, ventilator parameters, laboratory findings, and the use of steroid or immunomodulatory therapies were collected and analysed. Univariate and multivariate logistic regression analyses were performed to identify the potential risk factors and clinical outcomes associated with pulmonary barotrauma. **Results**: The medical records of 204 patients were included. The incidence of pulmonary barotrauma was 22.5%. Lower C-reactive protein (CRP) levels at ICU admission, lower FiO_2_ requirements during the first week of MV, a higher positive end-expiratory pressure (PEEP) during the second week, and a prolonged mechanical ventilation duration were significantly associated with pulmonary barotrauma (*p* = 0.039, 0.049, 0.021, and 0.036, respectively). Patients who developed pulmonary barotrauma experienced longer ICU stays (*p* = 0.006) and higher all-cause ICU mortality (*p* = 0.009). **Conclusions**: Lower CRP levels and a lower FiO_2_ requirements, a higher PEEP use, and longer ventilator days were the independent risk factors for pulmonary barotrauma in our study population, leading to a longer ICU stay and higher all-cause ICU mortality.

## 1. Introduction

COVID-19 infection is caused by the highly contagious SARS-CoV-2 virus, which led to a worldwide pandemic beginning in March 2020 [1,2]. In the early phase of infection, SARS-CoV-2 primarily targets nasal and bronchial epithelial cells and pneumocytes through its structural spike protein, which binds to the angiotensin-converting enzyme 2 (ACE2) receptor. This binding facilitates viral uptake, which is further promoted by the type 2 transmembrane serine protease (TMPRSS2) through ACE2 cleavage and the activation of the SARS-CoV-2 spike protein. As viral replication accelerates, the epithelial–endothelial barrier becomes compromised, allowing the infection of pulmonary capillary endothelial cells, and accentuating inflammation which triggers the infiltration of monocyte and neutrophil. These changes result in interstitial mononuclear inflammatory infiltrates and oedema, commonly presenting as ground-glass opacities on computed tomographic (CT) imaging [3]. Liu et al. [4] showed that 14.1% of COVID-19-infected individuals develop severe disease. A retrospective cohort study of 210 patients reported that 41.8% of those with severe COVID-19 progressed to acute respiratory distress syndrome (ARDS), necessitating mechanical ventilation [5]. Nevertheless, Xu et al. [6] noted higher rates of ARDS among COVID-19 patients with comorbidities.

Extrinsic positive end-expiratory pressure (PEEP) is often employed in mechanically ventilated ARDS patients to minimise lung atelectasis and thereby improve oxygenation [7]. Briel et al. [8] demonstrated improved survival in ARDS patients treated with higher PEEP levels, although this was associated with a higher risk of pulmonary barotrauma. Pulmonary barotrauma occurs when there is an alveolar rupture due to the unrelieved pressure differential between an unvented body cavity and the surrounding air–fluid interface or across a tissue plane, leading to air leakage into extra-alveolar tissue. Pneumothorax is the most common clinical manifestation of pulmonary barotrauma, though pneumomediastinum with or without pneumothorax, and subcutaneous emphysema may also occur. In COVID-19 patients requiring invasive mechanical ventilation, the incidence of pulmonary barotrauma has been reported to range from 9% to 33% [9,10,11,12,13,14,15,16]. Nonetheless, pneumothorax or pneumomediastinum have also been described to occur independently of pulmonary barotrauma [17,18].

Previous studies have shown higher incidences of pulmonary barotrauma in COVID-19 patients, compared with non-COVID conditions, alongside an increased mortality rate [11,19]. The identified risk factors include a younger age, elevated lactate dehydrogenase (LDH), and mechanical ventilation using a peak inspiratory pressure greater than 40–50 cmH_2_O, a higher PEEP, an increased tidal volume and minute ventilation, and prolonged hospitalization [9,20,21,22,23]. Additional risk has been associated with tracheal tears secondary to traumatic intubation, especially during emergency intubation, and elevated endotracheal cuff pressure [24,25]. High cuff pressure has also been observed among COVID-19 patients with severe ARDS who required mechanical ventilation in the prone position [26].

While studies have yet to establish a significant correlation between comorbidities, especially pre-existing lung pathologies, and pulmonary barotrauma [27,28,29,30], the increased occurrence of pulmonary barotrauma among mechanically ventilated COVID-19 patients in our centre highlights a critical gap in understanding. This observation underscores the need to investigate the incidence, contributing risk factors, and clinical outcomes of pulmonary barotrauma in this population. Therefore, this study aims to determine the incidence of this condition and identify the risk factors associated with its development in mechanically ventilated COVID-19 patients.

## 2. Materials and Methods

### 2.1. Study Design and Setting

This retrospective cohort study included medical records of all patients who were mechanically ventilated due to COVID-19 pneumonia, aged 18 years and above, and admitted to the COVID-19 intensive care unit (ICU) from January 2021 through June 2022.

### 2.2. Ethics

This study was approved by the Research Committee of the Department of Anaesthesiology & Intensive Care, Universiti Kebangsaan Malaysia (UKM) and conducted in accordance with the Declaration of Helsinki following approval from the institutional Ethics Research Committee, UKM (JEP-2022-120). Given the retrospective nature of this study, which utilised anonymised patient data extracted from ICU charts and laboratory records without any direct patient interventions, the requirement for informed consent was waived by the institutional ethics committee.

### 2.3. Inclusion and Exclusion Criteria

COVID-19 infection was confirmed via reverse-transcriptase–polymerase chain reaction (RT-PCR) and/or antigen rapid test kit (RTK-Ag), along with confirmed radiological imaging changes. Medical records of patients who developed pulmonary barotrauma before or within 24 h of ICU admission, had iatrogenic pneumothorax, were readmitted to the COVID ICU, or were mechanically ventilated for reasons other than respiratory failure due to COVID-19 were excluded from the study.

### 2.4. Data Collection

Medical records of the recruited patients were retrieved from the Health Information Department, the Caring Hospital Enterprise System (C-HEts, HCTM, Kuala Lumpur, Malaysia), and the Picture Archiving and Communication System (PACS, HCTM, Kuala Lumpur, Malaysia) Medweb radiology portal. Data collection extended from the time of COVID ICU admission until discharge, death, or a maximum of 28 days of COVID ICU stay, whichever occurred first. The collected data were grouped into two groups: patients who developed pulmonary barotrauma and those who did not develop this complication. The demographic profiles of patients, comorbid conditions, and vaccination status were recorded. Upon ICU admission, additional data collected included the Acute Physiology and Chronic Health Evaluation (APACHE) score, the stages of ARDS as defined by the Berlin definition, and initial laboratory investigations. ARDS severity was categorized as mild when the PaO_2_/FiO_2_ ratio was >200 mmHg to ≤300 mmHg, moderate when the ratio was >100 mmHg to ≤200 mmHg, and severe when the ratio was ≤100 mmHg, with PEEP or continuous positive airway pressure (CPAP) ≥5 cmH_2_O. Prescription of steroid and/or immunomodulator treatment, as well as occurrences of pulmonary embolism or pulmonary barotrauma throughout the ICU stay, were also collected by searching through their medical records. Pulmonary barotrauma was confirmed by reviewing evidence from chest X-ray (CXR, Shimadzu Malaysia, Petaling Jaya, Selangor, Malaysia), thoracic high-resolution computed tomography (HRCT, Toshiba Medical Systems, Pulau Pinang, Malaysia) imaging, or bedside lung ultrasound formal reports and/or by case note documentation.

All patients included in this study were managed according to the lung-protective strategies outlined in the ARDS.net guidelines to prevent ventilator-induced lung injury. Pulmonary barotrauma was considered to have occurred in a mechanically ventilated COVID-19 patient when pneumothorax, pneumomediastinum, and/or subcutaneous emphysema were present. Confirmation of pulmonary barotrauma was based on imaging reports from the CXR and HRCT thorax, as reviewed by the radiologist. The presence of a visible thin, white line (pleural line) with the absence of lung markings beyond the pleural line, a rim of gas around the edges of the lung, anterior to the pericardium, a ring around artery sign, a tubular artery sign, a continuous diaphragm sign, and subcutaneous emphysema were among the radiological features used to diagnose pulmonary barotrauma. Meanwhile, the presence of subcutaneous emphysema was confirmed clinically by palpating the affected area for crepitus under the skin. Bedside lung ultrasound (Fujifilm Sonosite, Petaling Jaya, Selangor, Malaysia) findings, including the presence of a lung point, and the absence of the lung sliding sign (or the presence of the barcode sign), were used by ICU consultants to support the diagnosis in patients suspected of pulmonary barotrauma. Types of pulmonary barotrauma were documented, and the first incidence was recorded as the onset of pulmonary barotrauma. In addition, the presence of organizing pneumonia, endotracheal cuff pressure, and the use of prone ventilation were also recorded.

Daily data on the highest peak airway pressure (Ppeak) were collected from day 1 to day 28 of COVID ICU admission (or until discharge or death), along with the corresponding fraction of inspired oxygen concentration (FiO_2_), inspiratory pressure (IP), positive end-expiratory pressure (PEEP), pressure support (PS), ventilation rate, minute ventilation (MV), and tidal volume (TV).

This data was further categorised into three periods, days 1–7, 8–14, and 15 and above, during the COVID ICU stay. All data collected were analysed for their association with the development of pulmonary barotrauma. The duration of COVID ICU stays, and all-cause ICU mortality rate were recorded as outcomes associated with pulmonary barotrauma. Missing or incomplete data were treated as dropouts in the analysis.

### 2.5. Statistical Analysis

A minimum sample size of 170 medical records was required to achieve 80% study power, a 95% confidence level, and an estimated 20% dropout rate. The calculations were performed using the Krejcie and Morgan formula for finite populations, based on an estimated total of 200 COVID-19 cases [31].

Data analysis was performed using SPSS for Windows, version 26.0 (IBM Corp., Armonk, NY, USA). Descriptive statistics were presented as mean ± standard deviation, median (interquartile range), or frequency (percentages) as appropriate. For between-group comparisons, the independent *t*-test was used for normally distributed continuous data, while the Mann–Whitney U test was applied for nonnormally distributed data. Categorical variables were analysed using the chi-square test or Fisher’s exact test, where appropriate.

Logistic regression (LR) analysis was performed to explore associations between pulmonary barotrauma and potential risk factors, including inflammatory biomarkers and treatment received. Variables with *p*-value < 0.200 in the univariate analysis were included in the backward LR variable selection process. Model fit of the final model was tested using the Hosmer–Lemenshow goodness of fit test, classification tables, and the area under the receiver operating characteristic (ROC) curve. *p*-value < 0.05 was considered statistically significant.

## 3. Results

A total of 389 patients with a confirmed COVID-19 infection were admitted to our COVID ICU during the study period. Among these, 75 patients were not mechanically ventilated and 79 were admitted for reasons other than respiratory failure despite having a concurrent COVID-19 infection. Additionally, four patients were below 18 years of age, and ten patients had developed barotrauma prior to ICU admission; these patients were therefore excluded from the study. Sixteen medical records contained incomplete data, and one patient opted to be discharged from the COVID ICU at their own risk; therefore, these medical records were excluded from the final analysis. As a result, 204 patient medical records were included for data analysis.

The incidence of pulmonary barotrauma among mechanically ventilated patients was 22.5%. The types of pulmonary barotrauma observed included subcutaneous emphysema (73.9%), followed by pneumomediastinum (65.2%), pneumothorax (52.2%), and pneumopericardium (8.7%). The demographic characteristics, APACHE scores, comorbidities, ARDS severity, and vaccination status are shown in Table 1. Most patients had pre-existing comorbidities, with hypertension and diabetes mellitus being the most common. Interestingly, patients who developed pulmonary barotrauma during their ICU stay were significantly less critically ill upon ICU admission and had a higher prevalence of hypertension compared to those who did not develop pulmonary barotrauma.

Our findings revealed that pulmonary barotrauma occurred more frequently among patients with moderate ARDS in Table 2. Notably, the majority of patients in our study cohort had moderate ARDS; however, there was no significant association found between ARDS severity and pulmonary barotrauma.

Patients who developed pulmonary barotrauma had significantly higher PEEP levels applied during the second week of mechanical ventilation and experienced a longer duration of ventilator support, as shown in Table 3.

Laboratory data collected on the day of ICU admission, along with the presence of organizing pneumonia (OP), pulmonary embolism (PE), and the administration of steroids and immunomodulators, are presented in Table 4. Interestingly, patients with significantly elevated CRP levels and those diagnosed with PE were less likely to develop pulmonary barotrauma.

A logistic regression analysis was performed to explore factors associated with pulmonary barotrauma. In the univariate analysis, a higher APACHE score on ICU admission, and the presence of hypertension and PE were associated with a lower risk of developing pulmonary barotrauma. In contrast, the presence of ARDS, higher PEEP levels during the second week of MV, and a prolonged ventilation duration were significantly associated with an increased risk of pulmonary barotrauma, as detailed in Table 5.

A multivariate analysis further indicated that patients with lower CRP levels upon ICU admission, higher FiO_2_ requirements during the first week of MV, higher PEEP use during the second week, and longer ventilator days independently increased the odds of developing pulmonary barotrauma.

Patients who developed pulmonary barotrauma had a significantly longer stay in the COVID ICU, by approximately 5 days (mean ICU stays in patients with pulmonary barotrauma 17.07 ± 10.53 days vs. 12.34 ± 7.26 days in patients without pulmonary barotrauma, *p* = 0.006). Furthermore, patients with pulmonary barotrauma were associated with a markedly higher all-cause ICU mortality rate compared to patients without pulmonary barotrauma (67.4% vs. 32.6%, *p* = 0.009).

## 4. Discussion

The limited understanding of this novel, yet rapidly evolving COVID-19 virus, together with the heterogenous findings from published studies, has, however, hindered the progression in identifying an ideal treatment. Pulmonary barotrauma has been closely associated with increased morbidity and mortality [32] due to hypoxia and hypotension secondary to obstructive shock in cases of tension pneumothorax. We found the incidence of pulmonary barotrauma to be 22.5%, which closely aligns with the finding of Belletti et al. [16], who reported a rate of 24.1%. A lower incidence was reported by Edwards et al. [33] (9.4%), while Kahn et al. [13] observed a higher rate (33.3%). Nonetheless, several studies have documented a relatively consistent incidence range of 13–14.8% [10,11,13,15,20,23]. Notably, elevated rates of pulmonary barotrauma were also observed during the previous SARS and MERS coronavirus outbreaks, ranging between 12% and 34%, raising the possibility that viral infections, specifically coronavirus infections, may pose a unique risk [29,30,32,34].

While pre-existing lung diseases are traditionally associated with the development of pulmonary barotrauma during mechanical ventilation, we found no significant correlation between underlying comorbidities and pulmonary barotrauma in our COVID-19 patient cohort, consistent with observations from other studies examining COVID-19 patients [13,15,17,35,36]. A notable finding in our study was that lower CRP levels on COVID ICU admission were associated with a higher risk of developing pulmonary barotrauma. Severe COVID-19 disease is characterized by a hyperinflammatory state with the release of multiple cytokines, including IL-6, suggesting that inhibiting IL-6 may be a potential therapeutic target. However, IL-6 also plays a key role in viral clearance and angiogenesis. Its inhibition may therefore be detrimental by hindering viral clearance and interrupting the tissue remodeling and repair process that prevents pulmonary barotrauma. Taha et al. [36] similarly reported an increased pneumothorax rate among recipients of Tocilizumab, an immunomodulator that was also prescribed in our patients. Despite that, we cannot exclude the possibility that barotrauma occurred later in the disease course, possibly after CRP levels had already declined. In addition, a steroid was prescribed to nearly all patients in our study, with higher doses given to those with severe hypoxemia, which potentially contributed to the lower CRP value obtained in our study.

Despite the implementation of lung-protective ventilation strategies for ARDS patients, studies on COVID-19 continue to demonstrate a higher incidence of pulmonary barotrauma among COVID-19 patients compared to non-COVID-19 patients who are ventilated [10,15,16,33]. In contrast to other studies that found no differences in ventilator settings, we observed that a lower FiO_2_ requirements during the first week of mechanical ventilation and higher PEEP levels during the second week were associated with an increased risk of barotrauma [14]. It has been postulated that the lung pathophysiology in COVID-19 may differ from that of typical pneumonia, with two distinct phenotypes, namely, the “L” and “H” types. However, we acknowledge that the association of a lower FiO_2_ requirements with barotrauma in our study was marginally statistically significant (*p* = 0.049) and might represent a chance finding. Therefore, this observation should be taken in the context of this limitation and requires validation in larger, prospective studies before any clinical recommendations can be made.

Virus-induced damage to the ACE2 receptors in endothelial and epithelial cells may impair the lung’s cellular repair mechanism, thereby exacerbating the alveolar injury [37,38]. Nevertheless, the interplay between cytokine production, PEEP, and tidal volume may worsen the alveolar injury by amplifying the inflammatory response, ultimately predisposing patients to pulmonary barotrauma [39]. This finding suggests that a deeper understanding of the state of the disease itself may contribute to a higher weightage in preventing pulmonary barotrauma than mechanical ventilator settings alone. Supporting this, several published studies have found no association between the ventilator settings and the incidence of pulmonary barotrauma [10,14,15,17,36]. Notably, Lemmers et al. [14] reported that the PEEP and plateau pressure were lower on the day pulmonary barotrauma occurred compared to the day of ICU admission. Nonetheless, the heightened respiratory drive in COVID-19 patients may induce a self-inflicted lung injury due to the heterogenous alveolar strain, contributing to the development of pulmonary barotrauma and explaining its frequent onset between days 8–14 of disease progression [40].

The duration of mechanical ventilation was also linked with an increased risk of pulmonary barotrauma, consistent with the findings by Taha et al. [36]. This aligns with the findings of a higher frequency of pulmonary barotrauma among mechanically ventilated patients compared with non-mechanically ventilated patients in other studies [16,29]. Nonetheless, mechanical ventilation itself can increase the risk of both macro- and microbarotrauma through factors such as positive pressure ventilation, recruitment maneuvers, coughing, and ventilator asynchrony [41,42]. Our study corroborates the previous research showing that patients who develop barotrauma experience longer ICU stays and higher mortality rates. Specifically, we observed a 67.4% mortality rate among patients with barotrauma, which aligns with the findings by Dubey et al. [12] and Kahn et al. [13].

This study has several limitations inherent to its retrospective design, including missing, misclassified, and non-retrievable data due to manual documentation by healthcare personnel. Future studies could benefit from a prospective, multi-centre design to enhance the generalizability of the results. Additionally, unmeasured confounding factors may have influenced our findings. Despite these limitations, our study explores the potential risk factors for pulmonary barotrauma and provides a valuable foundation for future research. These findings may also help clinicians in optimizing the care of critically ill COVID-19 patients.

## 5. Conclusions

Pulmonary barotrauma occurred in nearly a quarter of our COVID-19 ICU patients. The independent risk factors identified in our study included lower CRP levels, a lower FiO_2_ requirements during the first week of mechanical ventilation, a higher PEEP prescription during the second week, and a longer duration of ventilation. These factors were associated with prolonged ICU stays and significantly elevated mortality rates. Our findings underscore the importance of a deeper understanding of disease pathophysiology and the need for vigilant patient management to mitigate the risk of pulmonary barotrauma in patients with COVID-19.

## Figures and Tables

**Table 1:** Demographic, APACHE score, comorbidities, ARDS grade, and vaccination status.

	Pulmonary Barotrauma		*p* Value
	No (n = 158)	Yes (n = 46)	
**Age (year)**	57.06 ± 14.60	52.80 ± 14.27	0.082 ^a^
**Gender**			
Male	83 (52.5)	28 (60.9)	0.318 ^b^
Female	75 (47.5)	18 (39.1)	
**Race**			
Malay	82 (51.9)	24 (52.2)	0.362 ^c^
Chinese	52 (32.9)	16 (34.8)	
Indian	14 (8.9)	1 (2.2)	
Others	10 (6.3)	5 (10.9)	
**APACHE**	20.35 ± 7.97	16.87 ± 9.27	**0.013 ^a^**
**Hypertension**	107 (67.7)	22 (47.8)	**0.014 ^b^**
**Diabetes Mellitus**	79 (50.0)	18 (39.1)	0.194 ^b^
**Dyslipidaemia**	41 (25.9)	9 (19.6)	0.370 ^b^
**Asthma**	11 (7.0)	4 (8.7)	0.749 ^c^
**CAD**	19 (12.0)	5 (10.9)	0.830 ^b^
**CKD**	25 (15.8)	7 (15.2)	0.921 ^b^
**CHF**	6 (3.8)	2 (4.3)	>0.950 ^c^
**CVD**	10 (6.3)	6 (13.0)	0.207 ^c^
**Others**			
None	148 (93.7)	41 (89.1)	0.341 ^c^
Autoimmune	6 (3.8)	2 (4.3)	
Cancer	4 (2.5)	3 (6.5)	
**ARDS grade**			
Mild	29 (18.4)	3 (6.5)	0.095 ^b^
Moderate	96 (60.8)	35 (76.1)	
Severe	33 (20.9)	8 (17.4)	
**Vaccination**			
None	40 (25.3)	14 (30.4)	0.255 ^c^
First dose	18 (11.4)	10 (21.7)	
Second dose	29 (18.4)	6 (13.0)	
Booster	6 (3.8)	0 (0.0)	
Unknown	65 (41.1)	16 (34.8)	

Data is expressed as mean ± standard deviation or number (%). ^a^ Independent sample *t*-test; ^b^ Pearson chi-squared test; ^c^ Fisher Exact test. *p* value < 0.05 denotes statistical significance. CAD: coronary artery disease; CKD: chronic kidney disease; CHF: chronic heart failure; CVD: cerebrovascular disease; APACHE: Acute Physiology and Chronic Health Evaluation; ARDS: acute respiratory distress syndrome.

**Table 2:** Association between ARDS grades with pulmonary barotrauma.

	Pulmonary Barotrauma		*p* Value
	No (n = 158)	Yes (n = 46)	
**ARDS grade**			
Mild	29 (18.4)	3 (6.5)	0.052 ^a^
Moderate	96 (60.8)	35 (76.1)	0.056 ^a^
Severe	33 (20.9)	8 (17.4)	0.603 ^a^

Data is expressed as number (%). ^a^ Pearson chi-squared test. *p* value < 0.05 denotes statistically significance.

**Table 3:** Ventilator parameters, ETT cuff pressure, prone ventilation, and duration of ventilation.

	Pulmonary Barotrauma		*p* Value
	No (n = 158)	Yes (n = 46)	
**Ppeak (cmH_2_O)**			
D1–D7	29.0 (26.5, 31.0)	29.5 (27.5, 31.0)	0.252 ^b^
D8–D14	29.5 (27.5, 32.0)	30.0 (28.0, 32.3)	0.428 ^b^
D15–above	31.0 (28.3, 32.8)	28.5 (27.0, -)	0.591 ^b^
**IP (cmH_2_O)**			
D1–D7	15.0 (14.0, 18.0)	15.3 (14.0, 17.4)	0.952 ^b^
D8–D14	14.0 (12.0, 16.8)	15.3 (12.0, 18.0)	0.277 ^b^
D15–above	14.0 (12.0, 17.8)	18.0 (12.0, -)	0.497 ^b^
**FiO_2_ (%)**			
D1–D7	58.50 ± 18.53	63.41 ± 17.27	0.117 ^a^
D8–D14	58.93 ± 22.56	58.98 ± 12.60	0.993 ^a^
D15–above	63.96 ± 25.58	63.13 ± 17.96	0.949 ^a^
**PEEP (cmH_2_O)**			
D1–D7	10.0 (10.0, 12.0)	11.0 (10.0, 12.0)	0.208 ^b^
D8–D14	10.0 (8.0, 12.0)	11.0 (10.0, 12.0)	**0.024 ^b^**
D15–above	10.0 (8.0, 12.0)	10.0 (8.0, 12.0)	0.948 ^b^
**Rate (breath/min)**			
D1–D7	16.21 ± 2.64	16.24 ± 2.05	0.941 ^a^
D8–D14	16.48 ± 3.78	15.91 ± 2.81	0.513 ^a^
D15–above	16.62 ± 3.51	20.25 ± 3.30	0.056 ^a^
**PS (cmH_2_O)**			
D1–D7	14.0 (12.0, 15.0)	14.8 (12.0, 16.0)	0.127 ^b^
D8–D14	12.0 (12.0, 15.0)	14.0 (12.0, 14.0)	0.758 ^b^
D15–above	12.0 (11.0, 15.0)	14.0 (11.8, 15.5)	0.525 ^b^
**TV (mL)**			
D1–D7	525.68 ± 102.25	498.95 ± 80.55	0.072 ^a^
D8–D14	553.75 ± 145.57	520.82 ± 75.77	0.138 ^a^
D15–above	530.07 ± 154.28	485.50 ± 111.17	0.536 ^a^
**RR (breath/min)**			
D1–D7	18.0 (16.0, 20.0)	18.5 (16.0, 21.0)	0.071 ^b^
D8–D14	20.3 (18.0, 24.0)	20.0 (18.0, 21.1)	0.465 ^b^
D15–above	21.5 (18.4, 24.3)	19.0 (18.0, 24.1)	0.560 ^b^
**MV (L/min)**			
D1–D7	8.4 (7.7, 9.5)	8.7 (7.5, 10.1)	0.631 ^b^
D8–D14	10.4 (8.9, 11.8)	8.8 (8.0, 11.2)	0.066 ^b^
D15–above	10.0 (8.4, 11.9)	9.8 (6.5, 11.9)	0.721 ^b^
**ETT cuff pressure (cmH_2_O)**			
D1–D7	26.0 (26.0, 26.0)	26.0 (26.0, 26.0)	0.794 ^b^
D8–D14	26.0 (26.0, 26.0)	26.0 (26.0, 26.0)	0.117 ^b^
D15–above	26.0 (26.0, 26.0)	26.0 (26.0, 28.0)	0.409 ^b^
**Duration of ventilation (days)**	11.07 ± 7.53	14.98 ± 9.80	**0.004 ^a^**
**Prone**	59 (37.3)	21 (45.7)	0.310 ^c^

Data is expressed as mean ± standard deviation, median (IQR) or number (%). ^a^ Independent sample *t*-test; ^b^ Mann–Whitney U test; ^c^ Pearson chi-squared test. *p* value < 0.05 denotes statistical significance. D: day, ETT: endotracheal cuff, RR: patient respiratory rate; Ppeak: peak airway pressure; IP: inspiratory pressure; PEEP: positive end-expiratory pressure; PS: pressure support; TV: tidal volume; MV: minute ventilation.

**Table 4:** Biochemical, lung pathology, and the use of steroid and immunomodulator.

	Pulmonary Barotrauma		*p* Value
No (n = 158)	Yes (n = 46)		
Lymphocyte (×10^9^/L)	0.80 (0.50, 1.03)	0.60 (0.50, 1.10)	0.418 ^a^
Platelet (×10^9^/L)	237.0 (194.5, 303.0)	266.0 (191.0, 327.3)	0.341 ^a^
CRP (mg/dL)	8.53 (3.84, 17.20)	5.87 (2.76, 11.45)	**0.033 ^a^**
Ferritin (mcg/L)	1424.15 (767.62, 3049.85)	2035.16 (1024.58, 3068.86)	0.222 ^a^
D-dimer (g/L)	2.11 (1.05, 3.99)	2.18 (0.82, 5.18)	0.788 ^a^
LDH (IU/L)	562.50 (440.75, 764.00)	680.00 (487.00, 890.00)	0.078 ^a^
OP	97 (61.4)	32 (69.6)	0.312 ^b^
PE	47 (29.7)	6 (13.0)	**0.023 ^b^**
Use of steroid	156 (98.7)	46 (100.0)	>0.950 ^c^
Use of immunomodulator	114 (72.2)	29 (63.0)	0.235 ^b^

Data is expressed as median (IQR) and number (%). ^a^ Mann–Whitney U test; ^b^ Pearson chi-squared test; ^c^ Fisher Exact test. *p* value < 0.05 denotes statistical significance. CRP: C-reactive protein; OP: organizing pneumonia; PE: pulmonary embolism; LDH: lactate dehydrogenase.

**Table 5:** Risk factors for pulmonary barotrauma.

	Univariate Analysis		Multivariate Analysis	
	OR (95% CI)	*p* Value	Adj. OR (95% CI)	*p* Value
**Age (year)**	0.98 (0.96, 1.00)	0.084		
**Gender**				
Male	Reference			
Female	0.71 (0.36, 1.39)	0.319		
**Race**				
Malay	Reference			
Chinese	1.05 (0.51, 2.16)	0.892		
Indian	0.24 (0.03, 1.95)	0.184		
Others	1.71 (0.53, 5.48)	0.368		
**APACHE**	0.95 (0.91, 0.99)	0.014		
**Hypertension**	0.44 (0.22, 0.85)	0.015		
**Diabetes Mellitus**	0.64 (0.33, 1.26)	0.196		
**Dyslipidaemia**	0.69 (0.31, 1.56)	0.377		
**Asthma**	1.27 (0.39, 4.20)	0.692		
**CAD**	0.89 (0.31, 2.54)	0.831		
**CKD**	0.96 (0.38, 2.37)	0.921		
**CHF**	1.15 (0.22, 5.91)	0.866		
**CVD**	2.22 (0.76, 6.48)	0.144		
**Others**				
None	Reference			
Autoimmune	1.20 (0.23, 6.19)	0.825		
Cancer	2.71 (0.58, 12.58)	0.204		
**ARDS grade**				
Mild	Reference			
Moderate	3.52 (1.01, 12.30)	0.048		
Severe	2.34 (0.57, 9.67)	0.239		
**Vaccination**				
None	Reference			
First dose	1.59 (0.59, 4.25)	0.357		
Second dose	0.59 (0.20, 1.72)	0.335		
Booster	-	-		
Unknown	0.70 (0.31, 1.59)	0.399		
**Lymphocyte (×10^9^/L)**	0.89 (0.53, 1.49)	0.653		
**Platelet (×10^9^/L)**	1.00 (1.00, 1.01)	0.262		
**CRP (mg/dL)**	0.97 (0.93, 1.01)	0.153	0.90 (0.81, 0.99)	**0.039**
**Ferritin (mcg/L)**	1.00 (1.00, 1.00)	0.801		
**D-dimer (g/L)**	1.00 (0.99, 1.01)	0.707		
**LDH (IU/L)**	1.00 (1.00, 1.00)	0.850		
**OP**	1.44 (0.71, 2.91)	0.313		
**PE**	0.35 (0.14, 0.89)	0.028	0.33 (0.08, 1.31)	0.114
**Prone**	1.41 (0.73, 2.74)	0.311		
**Ppeak (cmH_2_O)**				
D1–D7	1.04 (0.96, 1.13)	0.341		
D8–D14	1.05 (0.91, 1.22)	0.485		
D15–above	0.94 (0.70, 1.27)	0.699		
**IP (cmH_2_O)**				
D1–D7	0.99 (0.89, 1.09)	0.831		
D8–D14	1.02 (0.91, 1.15)	0.751		
D15–above	1.08 (0.85, 1.37)	0.534		
**FiO_2_ (%)**				
D1–D7	1.01(1.00, 1.03)	0.119		
D8–D14	1.00 (0.98, 1.02)	0.993	0.95 (0.90, 0.99)	**0.049**
D15–above	1.00 (0.96, 1.04)	0.948		
**PEEP (cmH_2_O)**				
D1–D7	1.10 (0.91, 1.34)	0.310		
D8–D14	1.37 (1.02, 1.84)	0.039	1.68 (1.08, 2.61)	**0.021**
D15–above	1.00 (0.63, 1.59)	>0.950		
**Rate (breath/min)**				
D1–D7	1.01(0.88, 1.15)	0.940		
D8–D14	0.95 (0.83, 1.10)	0.510		
D15–above	1.35 (0.97, 1.87)	0.072		
**PS (cmH_2_O)**				
D1–D7	1.09 (0.96, 1.25)	0.192		
D8–D14	1.04 (0.86, 1.26)	0.664		
D15–above	1.18 (0.76, 1.84)	0.458		
**TV (mL)**				
D1–D7	1.00 (0.99, 1.00)	0.113		
D8–D14	1.00 (0.99, 1.00)	0.304		
D15–above	1.00 (0.99, 1.00)	0.528		
**RR (breath/min)**				
D1–D7	1.09 (0.98, 1.21)	0.115		
D8–D14	0.95 (0.84, 1.07)	0.398		
D15–above	0.94 (0.73, 1.20)	0.599		
**MV (L/min)**				
D1–D7	1.04 (0.85, 1.26)	0.724		
D8–D14	0.83 (0.67, 1.04)	0.106		
D15–above	0.92 (0.64, 1.31)	0.640		
**ETT cuff pressure (cmH_2_O)**				
D1–D7	0.73 (0.21, 2.57)	0.626		
D8–D14	0.27 (0.03, 2.62)	0.261		
D15–above	2.02 × 10^14^ (0.00, ∞)	0.998		
**Duration of ventilation (days)**	1.05 (1.02, 1.10)	0.006	1.08 (1.01, 1.16)	0.036
**Use of steroid**	-	0.999		
**Use of immunomodulator**	0.66 (0.33, 1.32)	0.237		

The *p* value < 0.05 denotes statistical significance. Variables with *p*-value < 0.200 in the univariate analysis were included in the variable selection process using backward LR method. CAD: coronary artery disease; CKD: chronic kidney disease; CHF: chronic heart failure; CVD: cerebrovascular disease; APACHE: Acute Physiology and Chronic Health Evaluation; ARDS: acute respiratory distress syndrome. D: day, ETT: endotracheal cuff, RR: patient respiratory rate; Ppeak: peak airway pressure; IP: inspiratory pressure; PEEP: positive end-expiratory pressure; PS: pressure support; TV: tidal volume; MV: minute ventilation.

## Data Availability

The data is contained within the article.

## Abbreviations

APACHE	Acute Physiology and Chronic Health Evaluation
ARDS	Acute Respiratory Distress Syndrome
CAD	Coronary artery disease
CKD	Chronic kidney disease
CHF	Chronic heart failure
CRP	C-reactive protein
CT	Computed tomographic
CPAP	Continuous positive airway pressure
CVD	Cerebrovascular Disease
D	Day
ETT	Endotracheal cuff
HRCT	High-resolution computed tomography
IP	Inspiratory pressure
RR	Patient respiratory rate
Ppeak	Peak airway pressure
OP	Organizing pneumonia
LDH	Lactate dehydrogenase
MV	Minute ventilation
PEEP	Positive end-expiratory pressure
PE	Pulmonary embolism
PS	Pressure support
TV	Tidal volume
